# Supplementary material for: A Phenomenological Inquiry of the Shift to Virtual Care Delivery: Insights from Front-Line Primary Care Providers
Source: Healthcare (Basel). 2024 Apr 20;12(8):861. doi: 10.3390/healthcare12080861 (PMC11050693; doi:10.3390/healthcare12080861)
Supplement: Supplementary file 1 [file healthcare-12-00861-s001.zip › healthcare-2868704-supplementary.pdf]

A Phenomenological Inquiry of the Shift to Virtual Care Delivery: Insights from Front-Line Primary Care Providers

Authors: Gayle Halas [1,*], Alanna Baldwin [1], Lisa LaBine [2], Kerri MacKay [2,3], Alexander Singer [2] and Alan Katz [2,4]

Supplementary Material Additional Participant Verbatim Quotations (as per themes)

Themes	Participant Quotations
Technology & Basic Tools Required	➢ *...many [patients] don't have a phone. So for those folks, they come in and they actually use our clinic phone. So they're in the exam room and talking to us at home. So they're in the clinic and we're at home. (FG Participant 601)*<br><br>➢ *Most of my patients are getting a call from my cell phone. So it does say my name and I'm okay with that. I've known these people long enough that they respect not calling me back or misusing my cell phone number… but that's a very individual thing in our clinic. (FG Participant 401)*<br><br>➢ *I know how that goes a lot of times…getting [the technology] set up and trying to explain it to people. And if they're not technologically advanced, it's like, 'well, how do I do this? And what do I click?' .....I didn't find that there would be that much benefit for the cost of how much setup and hassle it would be. (FG Participant 103)*<br><br>➢ *...a lot of this stuff we've learned on the fly, like the rest of the pandemic. So having some education around making use of the tools would be really important just like we do with the rest of our practice. So the other people that are doing it now have kind of figured it out on their own for the most part. (FG Participant 401)*<br><br>➢ *I think for fee for service positions, the cost is a barrier for some people with regards, like for physicians to implement the virtual care model. It's quite a bit more expensive. But I think that that's just the overhead piece of it, right, is expensive and but it may be better option for some kinds of calls. I know lots of patients would say, can I not just FaceTime you? But I didn't want to use my own personal device. So I think some people do need that face-to-face connection. But for fee for service, it's not cheap. I think it can add up when you're already looking at all the other overhead costs. (FG Participants 602)*
The Essentials of Communication	➢ *there's like the art and there's the science of medicine, right? So from the science point of view I find that it's fairly straightforward to take a history and then if they have a UTI,*

	you can treat it empirically over the phone. Those things are fairly routine, easy, straightforward to deal with. It's the art component, which is a little more challenging, whether there's motivational interviewing or if it's helping them with some contexts, psychosocial issues or helping them along the process changes have changed to go from pre-contemplation to contemplation to action, all that. Those things are a lot easier to do with some body language connection, like a lot of communication is body language, right? And so that person missing when you were just strictly over the phone, whereas as you're doing telehealth or video chats like this, … your advantage [is] the way you communicate, your tone, your facial expressions, all of those things help a lot. So I think that's, what's missing when you use a phone system. (FG Participant 203)<br><br>➢ *…how different it feels on the phone. So particularly with mental health conversations, it's a bit awkward. Like, there are these pauses and then you both start to talk and there's no visual cues of who's about to say something or what the body language is… (FG Participant 201)*<br><br>➢ *I think one thing about the virtual visits is I find it's tremendously easier when it's one of my patients that I know, versus if I'm talking to one of my colleagues' patients who you don't know whether this is the person who minimizes things or is this the person who overreacts or you can't appreciate the subtle cues in their voices. For a lot of my patients, the way they're saying things will raise a flag for me. Just the way they're talking can make you realize, hey, there's something wrong here or there's something more serious maybe going on. (FG Participant 501)*<br><br>➢ *The other thing that I found a challenge with sometimes with a language barrier; if some patients needed an interpreter, it was sometimes hard to make sure I would understanding the problem correctly. (FG Participant 602)*<br><br>➢ *…any cognitive delay or cognitive decline patients are really difficult to see that way too. (FG Participant 201)*<br><br>➢ *And then also people who require a proctor or assistance to attend appointments. Those folks are hard to have a visit over the phone with even if they're proctor's present. (FG Participant 601)*
Workflow	➢ *We had our admin staff say to everyone that they would have a phone appointment to begin, and the doctor will decide if you*

	need to also come in like later today or tomorrow or so on, which did happen a lot. (FG Participant 201)<br><br>➢ *And at the very least, it ended up making an in-person visit much shorter, because you could do part of the history talking on the phone and then decide specifically what you needed to do for the physical exam. And then I would walk in, confirm that's exactly what we were doing. We did a pap, we walked out, it made it much faster. (FG Participant 303)*<br><br>➢ *Whereas in your setting XX, where you've got elderly people, getting them in, getting them out of the office, you've got to run two rooms and three rooms. You can just get off the phone and go to your next call. So as physicians, we can be more efficient. (FG Participant 102)*<br><br>➢ *I've had very positive experiences. People can get in to see me quickly, often the same day. (FG Participant 401)*<br><br>➢ *So if the person absolutely had to come in that day, we could make it happen [in Overflow Clinic]. But I would have to do that from one of the colleagues and say, listen, so-and-so needs to come in, I need to make this happen. So we trusted each other to recognize that. (FG Participant 101)*<br><br>➢ *And most of the time for those, it was actually appropriate that they came in 'cause it was for something that you had to see. So we would, I mean, for the most part, like if I was on the phone with someone and I said, okay, I have to see you, I wouldn't then say, okay, I'm going to hang up. I want you to phone back and make an appointment. I would say, do you want me to go into my schedule and make it right now or do you not know your schedule enough and do you prefer to call back or go online and do it? (FG Participant 501)*<br><br>➢ *So I think that a lot of you have mentioned that the appointment times are shorter. I mean, I agree with most of it, but sometimes the appointments have become a bit longer, especially when it consists of things that they need to describe. Now, if I were to see, for instance, a skin lesion, I know what I'm going to look for and I know he doesn't have to describe, like the patient wouldn't have to describe it for me. (FG Participant 603)*<br><br>➢ *Some of the forms we normally fill online but there are some portions of the form that need in-person evaluation, like pre-op forms for instance. We can take the history for the first initial piece of the form, but the rest, they have come in anyway. So, I*

	think that's sometimes a little bit more work. (FG Participant 603)<br><br>➢ *In my opinion, it is <u>more work</u> and <u>more stress</u> than actual visit because you need more follow-up, you need to rely on more on doing investigation. (FG Participant 302)*
Balancing Risk	➢ *I found the challenge that it's a lot of the times you can't do much over the phone if it becomes any more complicated than just simple stuff. So, especially at the beginning of the pandemic, it was sort of practicing a lot of defensive, maybe ordering tests that you wouldn't normally do if you were able to see them in the office. (FG Participant 604)*<br><br>➢ *Yeah, I have always, the last thing I always try to say to somebody is if you don't get better, let me know. And hopefully remembering to give them a timeframe, give this two weeks or whatever (FG Participant 202)*<br><br>➢ *I learned that you don't come out of a phone call usually with a diagnosis, but you've ruled out some things that like are dangerous and you come up with a plan on how you're going to like manage your follow-up. (FG Participant 303)*
Physician Work/Life Balance	➢ *For me personally, I've changed my schedule so many times this year to figure out like what's going to work. Honestly, and I can't even say that I figured it out totally. (FG Participant 303)*<br><br>➢ *[Virtual care options] have also made it really flexible to work from home or go to the clinic. So if you want to do each, whatever you have to do. (FG Participant 603)*<br><br>➢ *It's really nice not to have to get into work. It's a sketchy neighborhood that I work in so I'm not comfortable driving and parking and going out at the end of the day, particularly in the winter when it's dark. So, it's nice not to have to do that. (FG Participant 101)*<br><br>➢ *And we appreciated, we got an hour and a half extra every day, extra of time that we didn't have to drive. (FG Participant 301)*
Efficiency & Benefit	➢ *And I think a lot of people are contacting us for issues that they might not have, had they not had the option of doing a phone visit. (FG Participant 501)*<br><br>➢ *I was just going to say another population I think we talk a little too, maybe a little bit more often too, is the like sort of frail elderly person that like struggles to come in. So there I've got a number of people that will only come in when there is*

	into centimeter of snow on the ground and everything has to be perfect and it can't be raining and all this stuff 'cause they don't want to leave their apartment. They're afraid of falling or whatever. So I'm talking to those people a lot more too that I usually would talk to in the summer and then need to like, tee them up to until I can see them in the spring time. But they've been feeling okay to call through the winter with whatever issues they might have. (FG Participant 501)<br><br>➢ *…they were very agoraphobic or had mental health issues that really impeded their ability to get there in person, this was a godsend for them. We connected with all sorts of people last April. (FG Participant 101)*<br><br>➢ *I have people in communities that are, oh, half hour away and they don't want to drive all the way here just to hear about their cholesterol results or their A1C. I can take care of that over the phone. (FG Participant 202)*<br><br>➢ *It certainly has helped a lot of young mothers. They don't have to schlep kids in for their appointment. (FG Participant 101)*<br><br>➢ *For our population, the big one is transportation. So, lots of our patients do not show because they don't have bus fare. Their attendance rates actually greatly improved because they don't have to worry about trying to access bus fare. So, I think those would be the sort of major advantages for our group. (FG Participant 601)*<br><br>➢ *So that is a benefit. I mean, some of our people are really living with a lot of stress and a lot of chaos in their lives and I think I'm getting a better window into that. So that is a positive, even if the [virtual] visit just seems to be totally chaotic and it's still valid and valuable information. (FG Participant 101)*
